# Maternal and child predictors associated with loss to follow-up in the newborn hearing screening program: a cohort study in maternity hospitals in northeastern Brazil

**DOI:** 10.1590/2317-1782/20232022114

**Published:** 2023-09-08

**Authors:** Maria Helena Medeiros de Sá Lima Lucena, Hannalice Gottschalck Cavalcanti

**Affiliations:** 1 Programa associado de pós graduação em Fonoaudiologia Universidade Federal da Paraíba - UFPB - João Pessoa (PB), Brasil.; 2 Universidade Federal do Rio Grande do Norte - UFRN - Natal (RN), Brasil.; 3 Universidade Estadual de Ciências da Saúde de Alagoas - UNCISAL - Maceió (AL), Brasil.; 4 Departamento de Fonoaudiologia, Universidade Federal da Paraíba - UFPB - João Pessoa (PB), Brasil.

**Keywords:** Hearing Loss, Newborn, Neonatal Screening, Loss to Follow up Care, Risk Factor, Perda Auditiva, Recém-nascido, Triagem Neonatal, Assistência de Seguimento, Fator de Risco

## Abstract

**Purpose:**

Analyze maternal and child predictors associated with loss to follow-up in the newborn hearing screening program at maternity hospitals in northeastern Brazil.

**Methods:**

Retrospective cohort study, including secondary data from infants (n=604) referred to the newborn hearing screening program in two maternity hospitals for monitoring and/or diagnosis. The predictors evaluated included socioeconomic factors, such as maternal age, marital status, income, schooling, place of residence, number of children and number of prenatal visits. In addition, maternal and child health factors, such as smoking and drug intake during pregnancy, consanguinity, congenital infections, craniofacial malformations, use of ototoxic drugs, syndromes and a history of hearing loss in the family. Statistical analysis was performed based on binary logistic regression models, using the stepwise method.

**Results:**

The logistic regression model containing the number of prenatal visits and the history of hearing loss in the family was significant [χ2(2) =34.271; p<0.001]. The number of prenatal visits (OR = 2.343; 95% CI = 1.626 - 3.376) and family history of hearing loss (OR = 2.167; 95% CI = 1.507 - 3.115) were significant predictors. The other predictors were not significant.

**Conclusion:**

The results reveal that newborns whose mothers had ≤ 5 prenatal visits and those with a family history of hearing loss increased their likelihood of loss to follow-up by 2.3 and 2.1 times, respectively. It is important to provide subsidies for public health improvements in order to help advise, guide and educate mothers, especially during prenatal care.

## RESUMO

**Objetivo:**

Analisar preditores maternos e infantis associados a perda de seguimento do programa de triagem auditiva neonatal em maternidades em maternidades do nordeste do Brasil.

**Método:**

Estudo de coorte retrospectivo, incluindo dados secundários de lactentes (n=604) que foram encaminhados para o monitoramento e/ou diagnóstico do programa de triagem auditiva neonatal, em duas maternidades do nordeste do Brasil. Preditores avaliados incluíram fatores socioeconômicos, como, idade materna, estado civil, renda, escolaridade, local de residência, número de filhos e número de pré-natais; E fatores de saúde materno e infantil, como, fumo e drogas durante a gestação, consanguinidade, infecções congênitas, malformações craniofaciais, uso de drogas ototóxicas, síndromes e história de perda auditiva na família. Foi realizado análise estatística baseada em modelos de regressão logística binária, método *stepwise*.

**Resultados:**

O modelo de regressão logística contendo o número de pré-natais e a história de perda auditiva na família foi significativo [*χ*^2^(2) =34,271; p<0,001]. Foram preditores significativos, o número de pré-natais (OR = 2,343; IC 95%= 1,626 - 3,376) e a história de perda auditiva na família (OR =2,167; IC 95% = 1,507 - 3,115), os demais preditores, não apresentaram significância neste estudo.

**Conclusão:**

Os resultados demonstram que estão associados e aumentam as chances de perda de seguimento do programa, recém-nascidos cuja mães realizaram ≤ 5 pré-natais com 2,3 vezes mais chances e história de perda auditiva na família aumentando os riscos em 2,1 vezes a mais. É importante fornecer subsídios para que sejam implementadas melhorias na saúde pública que visem aconselhar, orientar e conscientizar as mães, principalmente durante os pré-natais.

## INTRODUCTION

It has been established that hearing loss can affect a child's ability to develop speech, language and social skills. The sooner children with hearing loss receive special care, the more likely they are to reach their full potential^(1)^.

The newborn hearing screening program (NHSP) in Brazil is part of a set of measures recommended by the Ministry of Health for comprehensive hearing healthcare in childhood, and is responsible for the early detection of hearing loss in newborns (NBs)^(1,2)^.

It is estimated that the prevalence of congenital hearing loss is between 1.7-11/1000 in live births^(3)^ and increasing by up to 10-fold when those with risk factors for hearing loss (RFHL) are included^(4)^.

According to World Health Organization (WHO)^(3)^ reports in the World Report on Hearing (WRH), untreated hearing loss represents an annual cost of over USD 980 billion. This includes expenses related to health, education, lost productivity, and social costs. Many of these expenditures could be mitigated using cost-effective interventions^(3)^.

NHSPs in Brazil follow the recommendations, guidelines and updates proposed by the Joint Committee on Infant Hearing (JCIH)^(4-6)^, with adaptations according to their social sanitary situation. The main recommendations that guide NHSPs are from the Multiprofessional Committee on Auditory Health (COMUSA)^(2)^, which discusses and endorses actions aimed at the hearing health of newborns, infants, preschoolers, schoolchildren, adolescents, adults and older adults^(7)^, in addition to the national guidelines proposed by the Ministry of Health^(1)^.

Reports on the effectiveness of NHSPs demonstrate disparity between developed and developing countries^(8-10)^. The JCIH proposes different quality indicators for these programs over time, one of which is related to the rate of adherence and losses of families at any stage of the program^(4,6)^. COMUSA always endorses and recommends quality indicators for the implementation and evaluation of comprehensive care actions for hearing health in childhood^(4,7)^. Although existing programs seek to comply with these, research has shown that they face significant difficulties due to the high dropout rates of families in the different phases of the NHSP^(9-13)^.

The literature describes several predictors for loss to follow-up in hearing screening programs, such as newborns who failed the newborn hearing test, younger mothers, remaining 5 days or more in the NICU, use of ototoxic medication, hearing loss in the family^(14)^, low socioeconomic level, unmarried parents and mothers with several children^(11)^.

Hearing Health Programs depend on the interrelationship of several factors, including support and public research policy, follow-up and monitoring of children, training of professionals at all levels of health care and the existence of centers of excellence for diagnosis and intervention^(1,4)^. In this respect, it is important to analyze the causes of loss to follow-up, covering every phase of the NHSP, which includes monitoring and diagnosis, providing assistance and information to create new strategies aimed at reducing these losses.

### Objective

Determine whether there is an association between maternal and/or child variables and loss to follow-up in the NHSP in maternity hospitals in the city of Natal/Brazil.

## METHODS

### Study design

The retrospective cohort study model was adopted, limited to the NHSP developed by two maternity hospitals in northeastern, Brazil, in the city of Natal, Rio Grande do Norte state. At the time of the study, these were the only maternity hospitals available for vaginal deliveries and low-risk pregnancies in the municipality. Free and informed consent was signed by all participants. The mean of all live births delivered in a hospital from 2007-2009 in Natal was 11.329,66.

Loss to follow-up in the NHSP is the outcome variable. Maternal and child predictors described below were included to investigate this variable. The inclusion criteria, social economic factors and risk indicators for hearing loss of all the variables selected were established by the Joint Committee on Infant Hearing (JCIH)^(6)^, based on evidence found in studies on universal NHSPs^(10,11,15,16)^.

The present study was approved by the Research Ethics Committee of the Universidade Potiguar (UNB) under protocol number 107/2007.

### Data source

In order to characterize the maternal and child variables, the database was systematized by extracting secondary data from the medical records of the maternity hospitals. The records contained information found on the prenatal medical charts.

All social economic and maternal and child health data were stratified to simplify analyses, as follows: socioeconomic data, such as maternal age, into two age groups: ≤ 25 years and > 25 years; marital status: married/common law or not; schooling: ≤ 4th grade and > 4th grade; family income, based on the monthly minimum wage at the time: up to 1 and more than 1; place of residence, categorized as those living in Natal or outside the city; number of children, classified as first-born, yes (only child) and no (second, third-born etc); prenatal visits, divided into: ≤ 5 and >5 s; and maternal and child health data, stratified into nominal categorical variables, with yes or no answers.

### Sample

The subjects of this study are mothers and infants born between July 2007 and March 2009. This cohort was accompanied until 2018.

**Figure 1:**
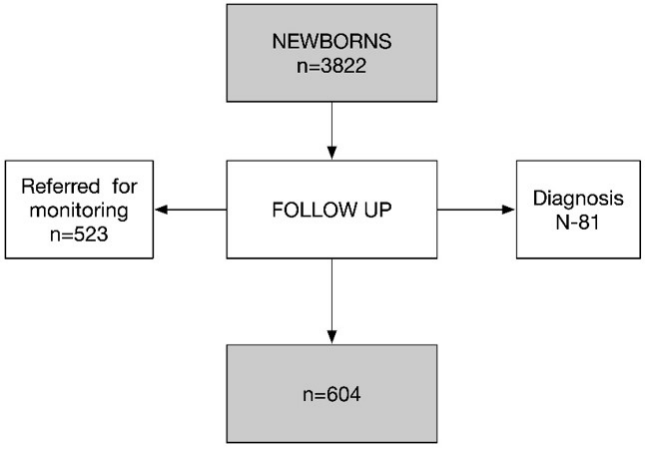
Flowchart of newborns referred for follow-up in the NHSP

In the database, live births (n=3822) at these maternity hospitals during the study period were analyzed. All newborns with life-threatening conditions transferred to intensive care units were excluded from the study, due to the assumption that health problems made them ineligible for the NHSP. All infants who underwent newborn hearing screening tests and who, based on the results, needed to be referred to the monitoring and/or diagnosis phase of the NHSP (n=604) were included (Figure 1).

Monitoring in the NHSP refers to those newborns and infants with risk indicators who responded satisfactorily during screening and who were referred for hearing evaluation every six months in he Specialized Rehabilitation Centers (SRC) by the Hearing Rehabilitation Service and in the High-Complexity Hearing Health Care Service of the Ministry of Health With respect to diagnosis, every newborn or infant who did not respond adequately during screening, was referred and diagnosed in the SRC as well^(1)^.

Newborns and infants without risk indicators who exhibited satisfactory responses during screening underwent monthly monitoring of hearing and language development in primary care^(1)^. These individuals were excluded from the study, as well as follow-up losses during the program that occurred before the monitoring and/or diagnosis phase. The term ‘loss to follow-up’ was specifically used for losses that occurred in the monitoring and diagnosis phases.

The infants underwent audiological monitoring or diagnosis at the SUVAG (Guberina’s Universal Verbotonal Auditory System) specialized care center, in the same state and municipality as the maternity hospitals under study. The SUVAG center is a philanthropic institution that has been promoting hearing health for 35 years, aimed at the prevention, diagnosis and rehabilitation of people with hearing and speech impairment, favoring their inclusion in society. The center provides weekly vacancies for newborns referred for hearing screening, thereby enabling early diagnosis. All the infants were submitted to the audiological diagnosis protocols recommended by international guidelines^(4,5)^, namely Brainstem Auditory Evoked Potential (BAEP), Transient and distortion product evoked Otoacoustic Emissions, Immittance measures and Visual Reinforcement Audiometry, in addition to follow-up with otolaryngologists and speech therapists. When hearing loss was identified, newborns were referred for auditory rehabilitation which included selection and adaptation of personal sound amplification devices (PSAD) and auditory speech language therapy.

### Statistics

In order to identify maternal and child predictors of loss to follow-up in the NHSP, descriptive statistical analysis and the association with variables of interest were performed for comparison with the outcome variable, using the χ^2^ association test, with Yates’ correction. All variables considered relevant for analysis were described using odds ratio (OR) values with respective confidence intervals (95%CI), evaluating the risks in relation to the independent variable of interest. Values were estimated by binary logistic regression models using the Stepwise method and p ≤ 0.05 was considered statistically significant. Prerequisites to apply binary logistic regression were the absence of both outliers and multicollinearity in the sample. Goodness of fit was assessed using the Hosmer-Lemeshow test. Statistical analyses were performed using the Statistical Package for the Social Sciences (SPSS), version 23.0.

## RESULTS

After hearing screening, 604 of the 3822 newborns were referred for follow-up care, 60.4% (n=365) of whom were lost to follow-up and 39.6% (n=239) monitored and/or diagnosed (Table 1). 34.6% from those referred to audiological diagnosis and 65.4% referred for audiological monitoring were lost to follow up.

**Table 1:** Distribution of absolute and relative frequencies of newborns who attended follow-up

***Variable***		***N***	***%***
NBs who attended follow-up	No	365	60.4
	Yes	239	39.6
**Total**			

**Caption:** NBS = Newborns; N = Number

Descriptive analysis of social economic factors (Table 2) demonstrated that most mothers (69.21%, n=380) were aged 25 years or younger, in a common law relationship (81.06%, n=445), had a schooling level above the 4th grade (83.87%, n=91), and a majority earned less than one minimum monthly wage (51.91%, n=285). With respect to place of residence, 83.87% (n=385) lived in Natal, for number of children, 56.64% (n=311) of the newborns were not the first child, and finally, prenatal visits showed a higher prevalence (57.55%, n=316) for more than 5 visits.

**Table 2:** Descriptive analysis of socioeconomic factors

***Variable***		***N***	***%***
**Marital status**	*Married*	445	81.06
	*Not married*	104	21.49
**Maternal age**	*≤ 25 years old*	380	69.21
	> *25 years old*	169	30.78
**Family income**	*≤1 monthly minimum wage*	285	51.91
	> *1 monthly minimum wage*	264	48.08
**Place of residence**	*Natal*	385	83.87
	*Outside Natal*	164	16.87
**Schooling**	*≤ 4th grade*	91	83.87
	> *4th grade*	458	16.12
**First-born**	*yes*	238	43.35
	*no*	311	56.64
**Number of prenatal visits**	*≤ 5 visits*	233	42.44
	> *5 visits*	316	57.55

**Caption:** N = Number

In regard to maternal and child health factors (Table 3), family history of hearing loss was the most prevalent hearing risk (43.5%) followed by TORCH infections (14.57%) and consanguinity (11.29%). Use of toxic drugs (11.11%) was mainly due to syphilis treatment. In Brazil all newborns in public maternity dwarfs undergo syphilis testing. All those with positive results are treated at the hospital, together with their parents, during 10 days.

**Table 3:** Descriptive analysis of maternal and child health factors following recommendations from JCIH^(6)^, COMUSA^(2)^ and Azevedo^(17)^

***Variable***		***N***	***%***
**Family history of hearing loss**	*yes*	239	43.53
	*no*	310	56.46
**Consanguinity**	*yes*	62	11.29
	*no*	487	85.89
**Congenital Infections (TORCHS)**	*yes*	80	14.57
	*no*	469	97.91
**Craniofacial malformations**	*yes*	10	1.82
	*no*	539	89.83
**Use of ototoxic drugs**	*yes*	61	11.11
	*no*	488	98.39
**Syndromes**	*yes*	8	1.46
	*no*	541	92.80
**Smoking during pregnancy**	*yes*	42	7.65
	*no*	507	95.30
**Drug use during pregnancy**	*yes*	25	4.55
	*no*	524	95.44

**Caption:** TORCHS = Toxoplasmosis, rubella, cytomagalovirus, herpes

In the relationship between the dependent variable and covariables, bivariate analysis of socioeconomic, maternal and child health factors demonstrated that the number of prenatal visits [χ2(1) = 17.839; p<0.00] with estimated risk (OR = 2.18; 95% CI = 1.525 - 3.116) and family history of hearing loss (HL) [χ2(1) = 15.268; p<0.001] with estimated risk (OR = 1.991; 95% CI = 1.417 - 2.799) were statistically significant and included in the logistic regression models, while the remaining factors were not statistically significant. Risk factor selection was based on JCIH^(6)^, COMUSA^(2)^ and Azevedo^(17)^.

Logistic regression analysis was conducted to determine whether the number of prenatal visits and family history of hearing loss (maternal and child health factor) are predictors of loss to follow-up in the hearing screening program.

The independent variables analyzed met the prerequisite of multicollinearity with tolerance > 1 and VIF (Variance Inflation Factor) < 10.

The model (Table 4) containing the number of prenatal visits and family history of hearing loss was also significant [χ2(2) = 37.151; p<0.001]. The number of prenatal visits (OR = 2.343; 95% CI = 1.626 - 3.376) and family history of hearing loss were significant predictors (OR = 2.167; 95% CI = 1.507 - 3.115); the other predictors analyzed were not significant. The model was more robust, explaining up to 64.7% of cases.

**Table 4:** Logistic regression model to predict maternal and child predictors associated with loss to follow-up in the NHSP in maternity hospitals in northeastern Brazil

***Variable***		***gl***	***p***	***OR***	***95%CI***	***p***	***OR***	***95%CI***
				Gross N=604			Adjusted N=555	
Number os prenatal visits (1)	≤ 5 visits (1)	1	.000	2.180	1.525 - 3.116	.000	2.343	1.626 - 3.376

Family history of HL (1)	yes (1)	1	.000	1.991	1.417- 2.799	.000	2.167	1.507 - 3.115

constant	-1.398	1				.000	.247	
Overall percentagem of the model	64.7%							

**Caption:** (1) = Reference category; gl = Degrees of freedom; p ≤ 0.05 (significance level); OR = Odds ratio; CI = Confidence interval

The Hosmer - Lemeshow test (p=0.902) was used to determine the degree of accuracy and whether there were significant differences between the classifications predicted by the model and those observed in practice. Since no significance was found, the alternative hypothesis was rejected, where observations would be different, and the null hypothesis accepted, where there are no significant differences between reality and the model, indicating reliability of the model.

Thus, the results reveal that a variable number of prenatal visits is a risk factor for loss to follow-up in the NHSP, where the likelihood of mothers who had up to 5 prenatal visits abandoning the program is 2.3 times greater than that of mothers with more than 5 visits.

A variable family history of hearing loss is also a risk factor for loss of follow-up, where the chances of an infant not adhering to the program are 2.1 times greater than those with no such history.

## DISCUSSION

The quality of a newborn hearing screening programs depends substantially on the number of babies who attend follow-up visits, whether for diagnosis or hearing monitoring. Loss to follow up still occurs in all stages of NHSPs, although an improvement in screening coverage and methods can be observed. Family history of hearing loss and craniofacial anomalies are risk factors for congenital hearing loss in neonates from well being nurseries^(18-20)^. When these families do not adhere to hearing follow up programs the prevalence of hearing loss in newborns may be widely underestimated. A recent study shows that the improvement of early identification of hearing loss in newborns, increased the prevalence from 0.9 to 1.7/1000 infants screened^(21)^. Hearing loss triples in school aged children due to lost cases in NHS as well as late onset hearing loss^(22)^.

Factors that contribute to non-attendance are educational disparities, uninformed parents, distance between home and a hearing health care center, setbacks at work, unfavorable attitudes and not prioritizing the issue. The creation of a database and education and information for parents may reduce these problems^(15)^.

Socioeconomic factors such as income and low schooling levels were also obstacles to parent participation in the hearing screening program^(16)^. As described by parents from a community in South Africa, contributing factors for the family to return with the baby to the program are the friendly attitude of the audiologist and effective communication between the audiologist and the family. A reminder of the day and time to return was also considered positive reinforcement^(10)^.

The presence of a full-time coordinator in the NHSP also helps achieve greater adherence of babies who need follow-up compared to programs without a coordinator^(12)^.

Our study did not find that income, schooling level or place of residence were predictors of non-attendance to audiological follow-up, unlike most literature reports. The fact that the program takes place in a municipal reference center may explain the lack of discrepancy between the economic and educational levels of families. As such, these variables do not elucidate the non-commitment to follow-up. However, the number of prenatal visits was a risk predictor for not adhering to the NHSP. Mothers with ≤ 5 prenatal visits are twice as likely to be lost to follow-up compared to those with 5 or more visits; this predictor is related to socioeconomic status^(13)^. Few studies report this as a maternal predictor for loss to follow-up, but one study that used logistic regression identified lower maternal age, more than one child, mothers without prenatal care or few prenatal visits, and the use of illegal substances as risk factors^(15)^.

There are no literature reports that associate high risk indicators for hearing loss with loss to follow-up in hearing screening programs. Prolonged stay in the newborn intensive care unit (NICU) is the most cited risk indicator^(16,23)^. Our study indicates that there is twice as much risk for a baby with a family history of hearing loss for adhering to the follow-up. This seems contradictory, since a risk indicator should reinforce the need for constant assessment of hearing status. One explanation may be that parents do not perceive this risk as threatening, specially if the baby has passed the child hearing screening and if the speech therapist has not explained the importance of this finding.

Our analyses allows for reflection on NHSPs to ensure that no child is left uncared for. Health services should carry out rigorous monitoring and guidance for mothers, since the association between maternal and child predictors (hearing loss in the family and number of prenatal visits) increases the chances of loss to follow-up in the NHSP. Studies aimed at identifying the reasons for the low number of prenatal visits and for dropping out of the NHSP, despite the presence of a risk indicator for hearing loss, require robust quantitative and qualitative analyses, which are essential to solving the problems underlying non-participation in a Brazilian context.

The results demonstrate the need for greater involvement of professionals who work in NHSPs. They are often in direct contact with the families that this study identified as at risk of non-participation. The program would also benefit from a national database that would allow for monitoring, as seen in other countries^(24)^. This database could make it easier to track program development and provide valuable information on the effects that changes may have on the participation rate. A comprehensive database could also identify children who, for unknown reasons, were lost to follow-up.

### Strengths and limitations

The main strength of this study is the population-based study design, which makes it less vulnerable to selection bias because it reached the entire sample within the surveyed locations. However, as with all record-based data, we cannot rule out inaccurate information and missing data, which could have affected the results. Data quality is considered reliable; however, the present study retrospectively evaluated a sample of newborns in two maternity hospitals in northeastern Brazil, which means it does not represent the entire region and that other evidence is needed to confirm these predictors as risk factors. Thus, it is not possible to generalize the findings to the newborn population referred for follow-up at Brazilian NHSPs.

## CONCLUSION

The results reveal that newborns whose mothers had ≤ 5 prenatal visits and those with a family history of hearing loss increased their likelihood of loss to follow-up by 2.3 and 2.1 times, respectively. It is important to provide subsidies for public health improvements in order to help advise, guide and educate mothers, especially during prenatal care.
